# High epiregulin expression in human U87 glioma cells relies on IRE1α and promotes autocrine growth through EGF receptor

**DOI:** 10.1186/1471-2407-13-597

**Published:** 2013-12-13

**Authors:** Gregor Auf, Arnaud Jabouille, Maylis Delugin, Sylvaine Guérit, Raphael Pineau, Sophie North, Natalia Platonova, Marlène Maitre, Alexandre Favereaux, Peter Vajkoczy, Masaharu Seno, Andreas Bikfalvi, Dmitri Minchenko, Oleksandr Minchenko, Michel Moenner

**Affiliations:** 1 Inserm, LAMC, UMR 1029, Talence F-33400, France; 2 University Bordeaux, Talence F-33400, France; 3 Department of Neurosurgery, Charité, Virchow Medical Center, Humboldt University, Berlin, Germany; 4 University Bordeaux, Bordeaux F-33077, France; 5 Inserm, U862, Bordeaux F-33077, France; 6 CNRS, UMR5297, IINS, Bordeaux F-33077, France; 7 University Okayama, Okayama, Japan; 8 Palladin Institute of Biochemistry, National Academy of Science of Ukraine, Kyiv, Ukraine

## Abstract

**Background:**

Epidermal growth factor (EGF) receptors contribute to the development of malignant glioma. Here we considered the possible implication of the EGFR ligand epiregulin (EREG) in glioma development in relation to the activity of the unfolded protein response (UPR) sensor IRE1α. We also examined EREG status in several glioblastoma cell lines and in malignant glioma.

**Methods:**

Expression and biological properties of EREG were analyzed in human glioma cells *in vitro* and in human tumor xenografts with regard to the presence of ErbB proteins and to the blockade of IRE1α. Inactivation of IRE1α was achieved by using either the dominant-negative strategy or siRNA-mediated knockdown.

**Results:**

EREG was secreted in high amounts by U87 cells, which also expressed its cognate EGF receptor (ErbB1). A stimulatory autocrine loop mediated by EREG was evidenced by the decrease in cell proliferation using specific blocking antibodies directed against either ErbB1 (cetuximab) or EREG itself. In comparison, anti-ErbB2 antibodies (trastuzumab) had no significant effect. Inhibition of IRE1α dramatically reduced EREG expression both in cell culture and in human xenograft tumor models. The high-expression rate of EREG in U87 cells was therefore linked to IRE1α, although being modestly affected by chemical inducers of the endoplasmic reticulum stress. In addition, IRE1-mediated production of EREG did not depend on IRE1 RNase domain, as neither the selective dominant-negative invalidation of the RNase activity (IRE1 kinase active) nor the siRNA-mediated knockdown of XBP1 had significant effect on EREG expression. Finally, chemical inhibition of c-Jun N-terminal kinases (JNK) using the SP600125 compound reduced the ability of cells to express EREG, demonstrating a link between the growth factor production and JNK activation under the dependence of IRE1α.

**Conclusion:**

EREG may contribute to glioma progression under the control of IRE1α, as exemplified here by the autocrine proliferation loop mediated in U87 cells by the growth factor through ErbB1.

## Background

Malignant gliomas are highly aggressive tumors and their treatment still remains a challenging issue. The moderate efficacy of current clinical approaches underline the need for new therapeutic strategies [1]. Some of these focus on the inhibition of EGF receptors, collectively referred to as the ErbB/HER tyrosine kinase receptor family [2]. This receptor family comprises four related members, ErbB1 to ErbB4, which are bound and activated by a set of thirteen distinct EGF-related peptide growth factors [2].

Amplification of ErbB1 and alteration of its activity are important contributors to glioma development [3,4]. For these reasons, phase II trials for high-grade gliomas have been targeting ErbB1 by using either humanized antibodies directed against the receptor extracellular domain (cetuximab, trade name Erbitux®), or pharmacological inhibitors of its protein kinase activity (erlotinib, gefinitib) [1,3,4]. The participation of the three others EGF receptors (ErbB2-ErbB4) in glioma progression by deregulation of ErbB signaling networks has also been considered [4-7].

The possible involvement of the EGF-like growth factors in glioma development was also questioned. An occasional increase of EGF, TGF-α or HB-EGF expression has been reported in malignant gliomas. Up-regulation of these growth factors may sustain autocrine loops [8-11] and contribute to tumor cell proliferation, invasion, survival and resistance to therapy [2,4].

EREG is a growth regulating peptide and a member of the EGF family mainly observed in placenta and peripheral blood macrophages in normal human tissues [12]. At the molecular level, EREG activates ErbB1 and ErbB4 homodimers as well as heterodimeric combinations of these two proteins and other EGF receptors [13,14]. EREG binds to ErbB1 with a lower affinity than EGF while exhibiting a higher mitogenic potential. This apparent inconsistency was explained by the prolonged stimulation of its receptors [13,15]. Because of its broad binding spectrum to ErbB proteins and high biological potency, EREG represents an influential activator of ErbB-dependent signaling networks in cancer. EREG is up-regulated in carcinoma cell lines [12] and is associated to the progression of breast, bladder and pancreatic carcinomas [16-18]. EREG is also an independent predictor of liver and lung metastasis in colorectal and bladder cancers, respectively [19,20].

To our knowledge, a single study considered EREG expression in glioma [21]. Previously, we showed that inhibition of the Unfolded Protein Response (UPR) sensor IRE1α (also named ERN1) down-regulated the expression of several pro-angiogenic growth factors in a glioma model [22]. Interestingly, the level of EREG transcripts was also strongly reduced in these conditions (GEO database, accession n° GSE22385), raising the hypothesis that its expression may be related to the endoplasmic reticulum (ER) physiology. Since EREG contributes to the angiogenesis process as well as to tumor metastasis in breast carcinoma models [23], we further considered its possible relationship to IRE1α and to glioma development and analyzed its status in several glioblastoma cell lines and in malignant glioma.

## Methods

### Reagents

Culture media were from Invitrogen (Cergy-Pontoise, France). Antibodies against ErbB1 were purchased from BD Biosciences (San Diego, USA). Anti-ErbB2 and anti-phospho-JNK (Thr183/Tyr185) were from Cell Signaling (Saint-Quentin-en-Yvelines, France). Anti-phospho-Tyr1173-ErbB1 was from Millipore (Molsheim, France). Anti-β-actin and anti-JNK antibodies were from Santa Cruz Biotechnology (Santa Cruz, USA). Recombinant EREG, monoclonal and polyclonal antibodies against EREG and control mouse monoclonal (isotype IgG_1_) antibodies were from R&D Systems (Minneapolis, USA). Secondary goat-anti-mouse antibodies coupled to biotin or to peroxidase were from DAKO (Trappes, France). Humanized anti-ErbB1 (Erbitux®, cetuximab) and anti-ErbB2 (Herceptin®, trastuzumab) antibodies were kindly provided by Merck Serono (Darmstadt, Germany) and by Roche (Mannheim, Germany), respectively. Primers are indicated in Additional file 1.

### Cloning

The dominant-negative IRE1 RNase mutant (IRE1Δ899; GenBank accession number JQ425696) was obtained by truncation of the carboxy-terminal 78 amino acids of IRE1α. The mutant was obtained by inserting a gatc motif at position 2812 of the BglII restriction site ^2799^tctgtcagagatc **“gatc”** tcctccgagccatgagaaataa^2833^. The frameshift insertion generates a stop codon 19 bases later. The wild type IRE1α amino acids sequence at positions 896–907 is –SVRDLLRAMRNK- and the C-terminal sequence of the mutant is –SVRDRSPPSHEK-COO^–^. The final sequence was controlled by DNA sequencing and was cloned in a pcDNA3 plasmid before transfection in U87wt cells and selection at 800 μg/ml G418.

### Cell culture

U87-MG (U87wt) cells were from ATCC (HTB-14). SF126 and SF188 cells were kindly provided by Dr. M. Czabanka (Charité Universitätsmedizin, Berlin). Cells were grown at 37°C, 10% CO_2_ in DMEM, 4.5 g/l glucose supplemented with 10% FBS, L-glutamine and antibiotics. Empty plasmid U87 (U87Ctrl) cells, U87 IRE1dn (U87dn) cells [22] and U87 IRE1Δ899 (U87Δ899) cells were grown in the presence of 500 μg/ml G418 and were used at passages 8–13 after transfection. The immortalized human astrocyte NHA/TS cell line and its tumorigenic NHA/TSR counterpart were kindly provided by Drs K. Sasai and S. Tanaka and were grown as reported [24].

### Proliferation and migration assays

Proliferation assay was performed in 96-well plates with DMEM containing 1% FCS and 30 ng/ml EREG. Serial propagation of cells in the absence of serum was developed as previously reported [25]. Briefly, cells were plated at 10 000 cells/cm^2^ in fibronectin-precoated 24-well plates. The serum-free complete medium consisted of a 1 to 1 mixture of DME/F12 medium, 1 mg/ml fatty-acid free BSA, 50 μg/ml high-density lipoproteins, 5 μg/ml transferrin, 5 μg/ml insulin with or without 10 ng/ml EREG. The medium was renewed every 3 days and cells were passaged after 9 days of culture. Cells were counted by using a cell counter (Coultronics, Margency, France). The transwell migration assays was performed as described previously [22]. Results were analyzed after counting of at least 15 fields of 150 μm^2^ each per condition and by three independent investigators.

### Immunoblot analysis

Subconfluent cells were lysed at 4°C with 100 mM Tris–HCl pH 7.5, 150 mM NaCl, 1 mM EDTA, 1 mM Na_3_VO_4_, 5 mM NaF, protease inhibitors (P8340; Sigma), SDS 1%. The cytosolic fraction was obtained by centrifugation for 2 min at 7000 rpm. After migration on SDS-PAGE, proteins were transferred to a nitrocellulose membrane and probed using antibodies against phospho- and total ErbB proteins, phospho- and total JNK proteins, β-actin or α-tubulin. Primary antibodies were revealed with a secondary HRP-antibody and detected by ELS Western bloting detection reagents (Amersham), or with a secondary antibody coupled to IRDye 800CW using the Odyssey infrared imaging system (Li-Cor Biosciences, Nebraska, US).

### ELISA against EREG

Conditioned media were obtained after a 16 h-incubation of cells in serum-free medium containing 1 mg/ml BSA. Proteins were precipitated in the presence of 80% ammonium sulfate, solubilized and dialyzed against PBS. A sandwich-type ELISA was developed for detection of human EREG using 3 μg/ml goat polyclonal antibodies for coating on 96-well plates and a mouse monoclonal anti-EREG (1 μg/ml) as the second antibody. Presence of EREG was indirectly measured using goat anti-mouse antibodies coupled to biotin and revelation was carried out using streptavidin peroxidase and the TMB substrate. Standard curves were obtained using recombinant hEREG and assays were performed in duplicate or triplicate. Measures were obtained with a SPECTRAmax spectrophotometer and calculations were developed from linear curves (r > 0.98).

### Gene expression analysis

Total RNAs extraction, real-time quantitative PCR (qPCR) and PCR analyses were carried out as previously described [22] using HPRT1, S16, α-tubulin and β-actin as reference genes. Experiments were performed in triplicate or tetraplicate from two or three independent cell cultures or from chicken and mouse tissues as indicated below. XBP1 splicing was monitored as reported before [22].

### Small interfering RNA knockdown experiments

U87 cells were plated at a density of 10^5^ cells per well in six-well plates. Small interfering RNA (siRNA) against human IRE1α (5′-GCGUCUUUUACUACGUAAUCU-3′) was from Eurofins MWG Operon (Ebersberg, Germany). ON-TARGETplus siRNA against XBP-1 (GCUCUUUCCCUCAUGUAUAC) and non-targeting siRNA (#D-001810-01-20) were from Dharmacon (Lafayette, CO). Transfection was performed for 48 h using lipofectamine RNAiMAX (Invitrogen) in accordance with the manufacturer’s protocol, with siRNA at a final concentration of 100 nM.

### Xenograft models

The Chorio-allantoic membrane (CAM) assay was developed as previously described [22]. At day 4 after implantation, tumors were excised from the CAM and pooled (n = 5 for each condition) before RNA extraction using Trizol reagent. Intracranial implantation was performed as follows: U87, SF126, SF188, NHA/TS and NHATSR cells were orthotopically implanted in 8–9 weeks of age RAG2/γ_c_ immunodeficient mice [22]. Cells (2.5x10^5^ cells, 3 μl) were implanted in the striatum of the left cerebral hemisphere, 0.1 mm posterior to bregma, 2.2 mm lateral and 3 mm in depth. For Kaplan–Meier survival analyses, 18 mice were implanted with U87Ctrl cells and half of them were treated by subcutaneous injection of 400 μg Erbitux® three times a week from day 4 to day 32 post-implantation. *In vivo* experiments were performed at the animal facility Université Bordeaux 1 (agreement n° B33-522-2) according to ethical criteria approved by the Ministère de l′Enseignement Supérieur et de la Recherche (MESR).

### Laser-capture microdissection

Tumors were xenografted in mice as described above. Brains were recovered at different times and frozen at −80°C. Tissue sections (30 μm) were obtained at −20°C using a CM3050 S microtome (Leica) and were mounted on PEN-membrane 1 mm glass slides (P.A.L.M. Microlaser Technologies AG, Bernried, Germany) that had been pretreated to inactivate RNase. Frozen sections were fixed by incubation for 1 min in pre-cooled (−20°C) 80% ethanol and stained with H&E for 30 s. Sections were then rinsed with RNase-free water for 30 s, dehydrated in a series of pre-cooled ethanol baths (30 s in 50%, 70% and 1 min in 100%) and air-dried. Immediately after dehydratation, LCM was performed using a PALM MicroBeam microdissection system version 4.0-1206 equipped with a P.A.L.M. RoboSoftware (P.A.L.M. Microlaser Technologies AG, Bernried, Germany). Microdissection was performed at 5X or 20X magnification. Total volumes of tumor tissues captured on one single cap were in the 0.8- to 8.7 x 10^6^ μm^3^ range and random areas were chosen within tumors. RNA samples with a RNA-Integrity Number (RIN) above 8 were kept for qPCR analyses after NanoDrop and Agilent validation. Three tumors were analyzed for each condition and qPCR were carried out in triplicates. Primers specifically recognized cognate human sequences and did not significantly cross-react with any mouse sequences as determined both in total mouse brain tissues and mouse brain sections obtained by LCM. Control qPCR were also performed from tumor tissues after omitting the reverse transcriptase step, giving no detectable signals after 40 complete run cycles.

## Results

### EREG expression in U87 glioma cells

**Figure 1:**
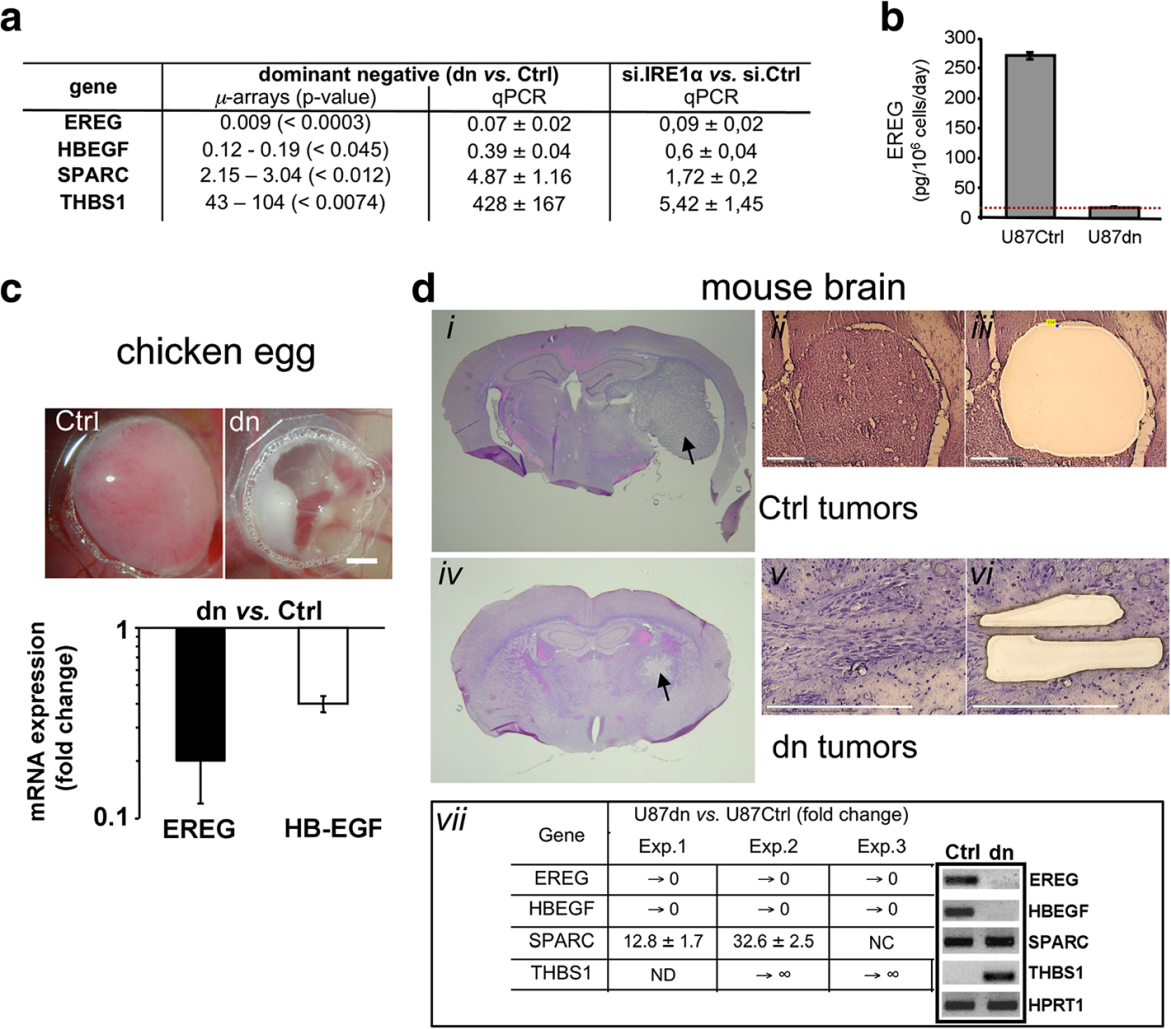
**Expression analyses of EREG and HB-EGF in IRE1α-deficient cells.** Analyses were performed using either the dominant-negative strategy (U87dn *vs.* U87Ctrl cells) or the siRNA IRE1α knockdown methodology. **(a)** Fold-increase in gene expression was examined from microarray data (GEO, #GSE22385) and by qPCR. For knockdown analysis, IRE1α siRNA-transfected U87wt cells were compared to nontargeting siRNA-treated cells (si.Ctrl). SPARC and THBS1 mRNA levels were given for comparison. qPCR mean values were ± SD. **(b)** EREG protein levels in cell-conditioned media as determined by ELISA. Results are mean values ± SD. The dotted line represents the limit of detection of the measure. **(c)** The chicken egg model. Cells were deposited onto the chicken CAM and tumors were allowed to grow for 4 days. Upper panel: microphotographs of U87Ctrl- and U87dn-derived tumors at day 4. Bar = 2 mm. Lower panel: variation of EREG and HB-EGF transcripts levels in U87dn *vs.* U87Ctrl tumors as measured by qPCR. Data are mean values of five pooled tumors ± SD. **(d)** Mouse model. Cells were intracranially implanted into the left frontal lobe and tumors were collected at d28 (U87Ctrl) and at d43 (U87dn) post-implantation. Brain sections were stained with H&E (*i*, *iv*). Aspect of tumors before (*ii*, *v*) and after (*iii, vi*) LCM (Bars = 300 μm). Tumor areas were dissected inside the tumor core in control animals (*ii*, *iii*) and multiple tumor cell bundles were collected in infiltrative dn tumors (*v, vi*). Gene expression analyses (*vii*) were carried out by qPCR using HPRT1 as reference. Results are fold-increase ± SD of triplicates in three independent experiments (Exp. 1–3). NC, no change; → 0, No Ct value obtained with U87dn tumors; → ∞, value > 3 000; ND, value could not be determined. Visualization of amplicons after 40 cycles of qPCR (panel *vii*, right).

Expression of EREG and HB-EGF, two members of the EGF family, was analyzed in U87 cells in culture conditions. Using transcriptome analysis, we observed that the two transcripts were abundant both in wild type U87 (U87wt) cells and in cells transfected with the empty vector (U87Ctrl cells), whereas ~100-fold (EREG) and 8-fold (HB-EGF) decreases were monitored in cells expressing an IRE1α dominant-negative protein (U87dn cells) (Figure 1a). Similar results were obtained by qPCR in independent cell cultures as well as in U87wt cells transfected with small interfering RNAs targeting IRE1α (si.IRE1α) (Figure 1a). Thus, both dominant-negative and siRNA knockdown approaches led to a significant decrease in EREG mRNAs in cells under-expressing IRE1α. As positive controls, SPARC and THBS1 genes were upregulated to different extents. Consistent values were obtained at the protein level by using an ELISA against EREG (Figure 1b). U87Ctrl cells released ~270 pg of diffusible EREG per million cells daily, whereas EREG immunoreactivity was undetectable in U87dn cell-conditioned media (< 20 pg per million cells per day).

Presence of EREG and HB-EGF mRNAs in U87 cells was also monitored in human tumor xenografts using the chicken chorio-allantoic membrane (CAM) and the mouse brain models. U87Ctrl and U87dn cells were implanted onto the CAM and tumors were grown for 4 days. Under these conditions, U87dn-tumors were small and merely avascular, compared to massive and angiogenic U87Ctrl-tumors (Figure 1c, upper panel) [22]. Tumors were then excised and total mRNA was extracted for qPCR analysis. EREG and HB-EGF mRNAs were present in smaller amounts (~5- and ~2.5-fold decreases, respectively) in U87dn-derived tumors as compared to U87Ctrl tumors (Figure 1c, lower panel). These transcripts were also quantified in the orthotopic glioma implantation model in mice using LCM coupled to qPCR analysis (Figure 1d). In these conditions, EREG and HB-EGF mRNAs were readily detected in U87Ctrl-derived tumors but not in U87dn-derived tumors (Figure 1d, panel *vii*). Thus, mRNA production of these growth factors occurred in an IRE1α-dependent manner in U87 glioma cells.

### EREG induced glioma cell proliferation and migration

**Figure 2:**
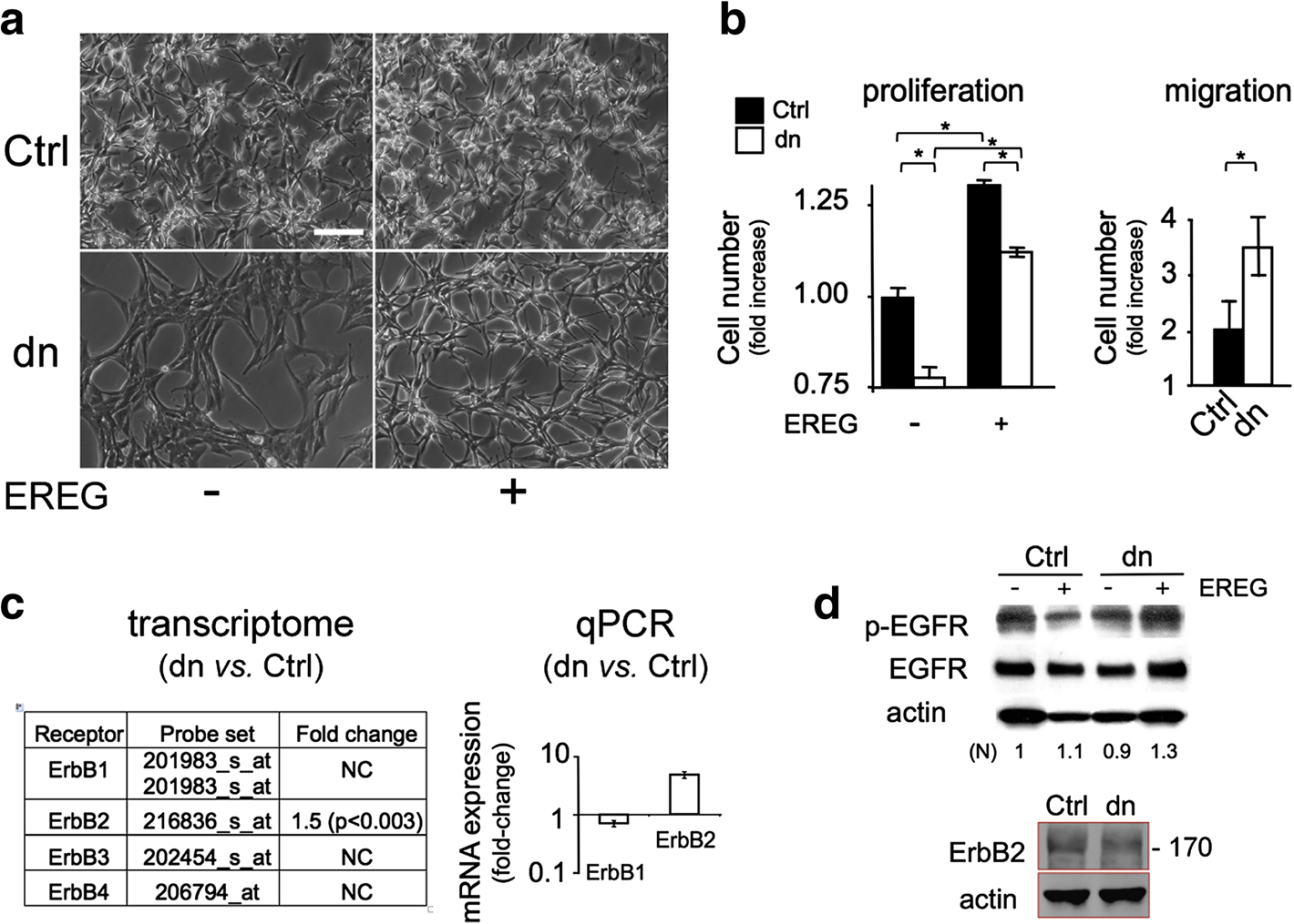
**Differential effects of EREG on morphology, growth and migration of U87Ctrl and U87dn cells. (a)** Morphological changes are selectively induced by EREG on U87dn cells. Cells were grown in the presence of 1% FCS with or without 30 ng/ml EREG. Photomicrographs of U87Ctrl and U87dn cells are shown after 3 days in culture. Bar = 50 μm. **(b)** Effects of EREG on U87 cell proliferation and migration. In the proliferation assay, cells were grown for four days. The total cell number was reported as fold-increase of the standard value (1.00) obtained with U87Ctrl cells in the absence of EREG. Results are the mean of triplicates ± SD. Mann–Whitney was performed for significance (*; p < 0.05). In the Transwell migration assay, cells were deposited in the migration chamber for 15 h and were then allowed to migrate for 9 h in the absence of serum, with or without EREG. Results were expressed as fold increase ± SD of the number of migrating cells in the presence *vs.* absence of EREG (*; p < 0.05). **(c)** EGF receptors are expressed in U87Ctrl and U87dn cells. Differential expression of ErbB1-4 mRNAs in U87dn *versus* U87Ctrl cells as depicted by transcriptomic (GEO, GSE22385; AffyID probe set numbers are indicated) and qPCR analyses. **(d)** Presence of EGFR (ErbB1) and ErB2 proteins in U87Ctrl and U87dn cells. For EGFR detection, cells were pre-incubated for 3 h in the absence of serum and were then stimulated or not with 30 ng/ml EREG for 20 min. Immunoblotting was performed using antibodies against EGFR, phospho-Tyr1173-EGFR (p-EGFR), ErbB2 or β-actin. Signal intensities of p-EGFR bands were quantified and normalized (N) to β-actin. The 1.0 value is used as the reference.

The effect of EREG on U87 cells was examined in cell cultures at low serum concentration. U87dn cells incubated for three days in the presence of EREG underwent notable scattering, which was not observed with U87Ctrl cells (Figure 2a). Such an effect has already been described using HeLa epithelial cells [15]. In addition to its morphological effect, EREG induced proliferation and migration of the two cell variants, these effects being more important in U87dn cells (Figure 2b). These results suggest the presence of functional ErbB proteins on the membrane of U87 cells.

Transcript and protein expression levels of ErbB1-4 were analyzed comparatively and quantitatively in the two cell types. EREG was reported to bind preferentially to ErbB1 and ErbB4, whereas ErbB2 does not bind any known ligand but contributes as a co-receptor to signal transduction [13,14]. Transcriptomic and qPCR analyses indicated that the respective amounts of ErbB1, ErbB3 and ErbB4 mRNAs are similar in the two U87 cell variants (Figure 2c), the level of ErbB3 transcript being almost undetectable. Besides, the amount of ErbB2 mRNA increased by ~1.5- to 4-fold in U87dn cells *vs.* U87Ctrl cells. Only ErbB1 and ErbB2 proteins were detected by immunoblotting (Figure 2d; data not shown), which is consistent with results reported by others in this cell model [6,26]. Finally, treatment of U87Ctrl and U87dn cells with EREG stimulated phosphorylation of the EGFR (ErbB1) protein at Tyr-1173 residue (~10% and ~40% increases in the two cell variants, respectively).

Next, we investigated the respective contribution of ErbB1 and ErbB2 to cell proliferation promoted by EREG. Cells were incubated in the presence of EREG under low-serum conditions, with or without inhibitory antibodies directed against either ErbB1 (Erbitux®) or ErbB2 (Herceptin®). As shown in Table 1, Erbitux® almost completely abrogated EREG-induced cell proliferation of U87Ctrl and U87dn cells, whereas Herceptin® had no significant effect. Thus, the effect of EREG on U87 cell proliferation was mediated mainly through ErbB1.

**Table 1:** Erbitux inhibits EREG-mediated proliferation of U87 cells

	**No antibody**	**Erbitux ® (anti-ErB1)**	**Herceptin ® (anti-ErB2)**
**U87Ctrl**	**130.1±4.4**	98.3±8.6(*)	**125.6±7.9 (ns)**
**U87dn**	**144.3±3.3**	104.3±3.9(*)	**131.6±8.8 (ns)**

Cells were plated at 7,500 cells/cm^2^ in 96-well culture dishes and were grown in the presence of 1% FCS in the presence or absence of EREG (30 ng/ml), Erbitux® (200 μg/ml; cetuximab) and Herceptin® (830 μg/ml; trastuzumab). Cells were counted in triplicate after four days of culture. EREG-induced cell proliferation was presented as mean percentage ± SD of the total cell number measured in the absence of EREG (100% reference value). Mann–Whitney was performed for significance: Erbitux® *vs.* no antibody (*; p ≤ 0.05); Herceptin® *vs.* no antibody (ns, not significant; p > 0.05).

In order to validate the existence of an EREG autocrine loop, a serial propagation of U87 cells was performed for four passages in a serum-free medium in the absence of growth factors. The culture medium was designed to allow better detection of endogenous growth promoting activities, including those of the EGF family [25]. Again, stimulation with EREG in these conditions resulted in a significantly higher growth rate of both U87Ctrl and U87dn cells (Table 2). This effect was reverted by adding either Erbitux® or anti-EREG antibodies. Interestingly, EREG blocking antibodies also consistently increased by 14% the U87Ctrl cell division time in the absence of exogenous EREG and this effect was not observed in U87dn cells under-expressing EREG. Thus, U87Ctrl cells, but not U87dn cells, actively stimulated themselves by producing both EREG and ErbB1.

**Table 2:** Autocrine loop induced by EREG in U87 cells

	**Division time in days (R value)**	
	**U87Ctrl**	**U87dn**
no treatment	2.73 (0.99)	5.08 (1.00)
EREG	2.20 (1.00)	2.86 (0.99)
EREG/Erbitux**®**	3.08 (1.00)	4.50 (1.00)
anti-EREG	3.12 (1.00)	4.76 (0.99)

Cells were plated at 10 000 cells/cm^2^ in 24-well plates and grown for four successive passages in serum-free condition in the presence or absence of 10 ng/ml EREG, with or without antibodies anti-ErbB1 (Erbitux®, 200 μg/ml) or anti-EREG (5 μg/ml). Cells were counted at each passage and division times were presented as best slopes obtained after four passages (29 days of growth) and from a series of triplicate experiments. Regression lines include the origin (R, correlation coefficient). Control mouse monoclonal antibody (isotype IgG1) had no significant effect.

The autocrine effect of EREG was then examined in a xenograft tumor model. After implantation of U87wt cells in mice brain, animals were treated for four weeks with or without Erbitux® and tumor aggressiveness was determined. As shown in Additional file 2, no significant effect of Erbitux® was evidenced in this experimental setting (see also ref. [27]), which may result of a limited antibody delivery to tumor tissues. Besides, the autocrine contribution of EREG is likely to be reduced in the U87 glioma model, as these fast-growing tumors secrete other growth-promoting and angiogenic polypeptides and may exploit alternative signaling pathways for expansion [22,28].

### EREG expression and glioma malignancy

**Figure 3:**
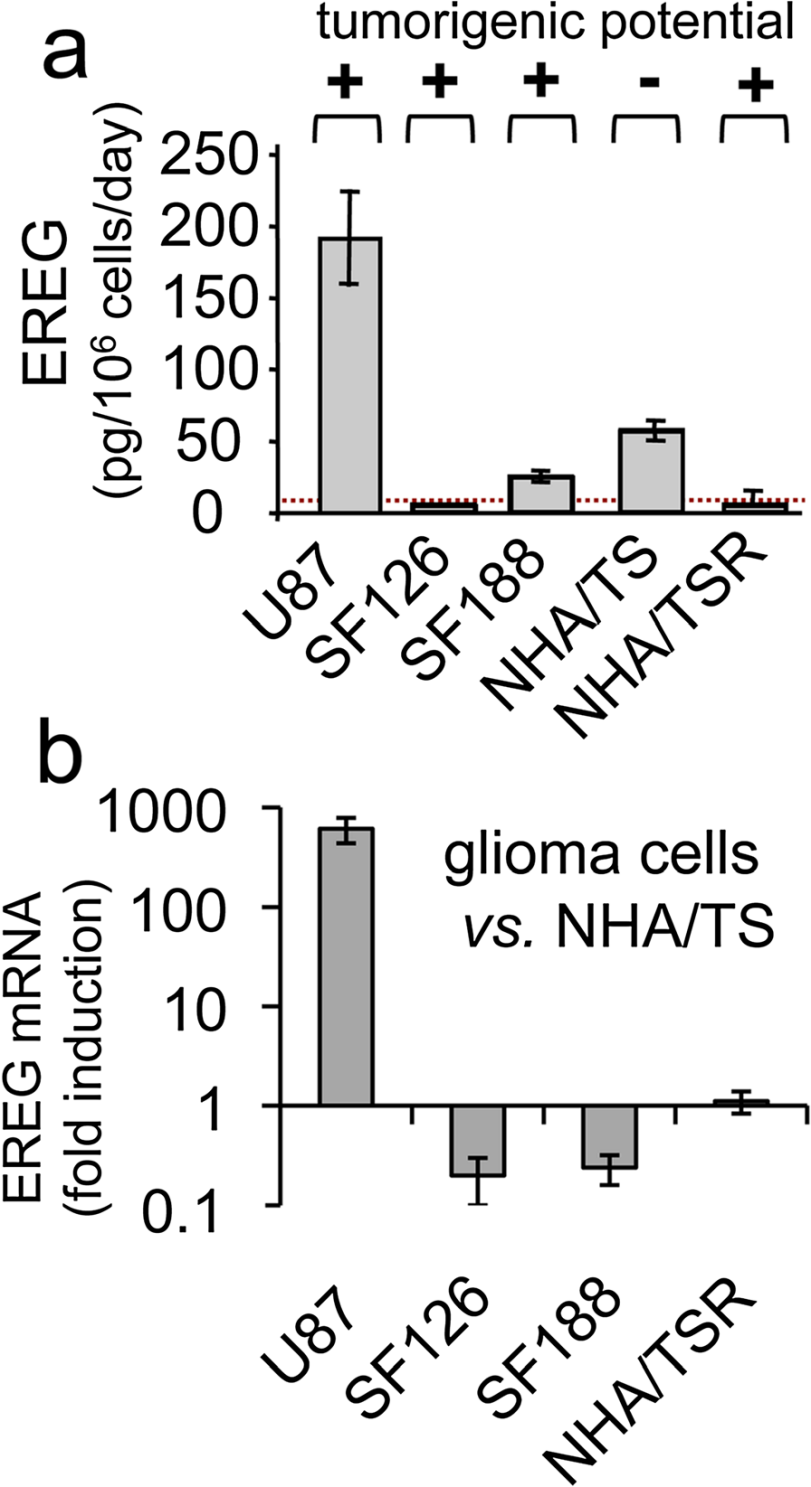
**Expression of EREG in human glioma cell lines. (a)** EREG immunoreactivity was measured by ELISA in culture media conditioned by glioblastoma cell lines (U87, SF126, SF188), immortalized/non-tumorigenic human astrocytes (NHA/TS) and the NHA/TS tumor variant expressing the *Hras* oncogene (NHA/TSR). The dotted line represents the limit of detection of the measure. The tumorigenic potential of each cell type was evaluated by immunohistochemistry after intracranial implantation of 250 000 cells and analysis of tumor progression at days 10, 20, 30 and 60 post-implantation. (+) tumorigenic, (−) not tumorigenic. **(b)** EREG mRNA expression was represented as fold induction in glioma cells *vs.* NHATS cells. qPCR was performed using HPRT1 as reference gene.

EREG mRNA and protein levels were monitored in several human glioma cell lines. As shown in Figure 3a, U87wt, SF126 and SF188 cells were highly tumorigenic in the orthotopic implantation model in mice and released highly variable amounts of EREG protein (up to 200-fold differences). Moreover, non-tumorigenic NHA/TS human astrocytes produced about five-times more EREG than their highly oncogenic *Hras*-transformed (NHA/TSR) counterparts. These results are consistent with those obtained at the mRNA levels (Figure 3b) and indicated that the release of EREG by these glioma cell lines did not strictly correlate with tumor malignancy.

We then evaluated the clinical significance of EREG expression in human gliomas, of which a significant percentage accumulates high levels of ErbB proteins. We documented EREG mRNA production by transcriptome mining using the Gene Expression Omnibus (GEO) and Oncomine databases (Additional file 3). Microarray analyses of gliomas at different grades of malignancy indicated that EREG transcripts were detected in highly variable amounts in tumor tissues, although no clear relationship was established between EREG mRNA levels and the glioma grade or brain tumor type. Individual cases presenting EREG upregulation were also observed by using PCR approaches in both anaplastic astrocytoma and glioblastoma, as compared to normal brain tissues [21].

### EREG expression in relation to IRE1α

**Figure 4:**
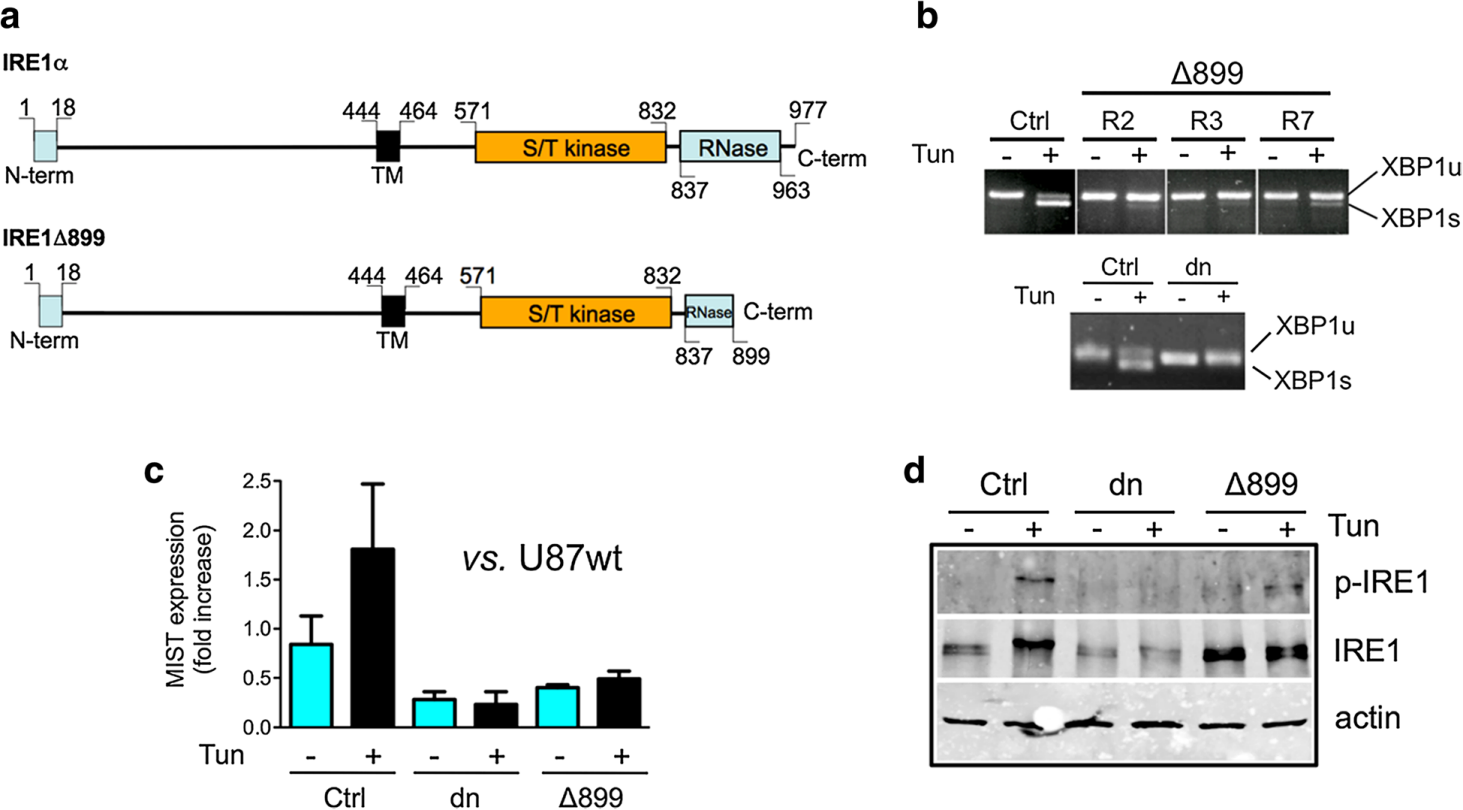
**Characterization of U87Δ899 IRE1 RNase dominant-negative cells. (a)** The U87Δ899 RNase construct was designed to express an IRE1α protein truncated at its cytoplasmic C-terminal end in the RNase domain. **(b)** Inhibition of XBP1 splicing in three different U87Δ899 RNase clones (R2, R3 and R7, upper panel) and in U87dn cells (lower panel). Cells were stimulated for 2 h with 10 μg/ml tunicamycin/DMSO (Tun) or with DMSO only. Amplification of XBP1 transcripts was carried out after reverse transcription using primers flanking the XBP1 mRNA splicing sites. PCR products were analyzed by electrophoresis on 2% agarose gels. XBP1s and XBP1u represent spliced and unspliced mRNA, respectively. **(c)** MIST transcripts were measured by qPCR in U87wt, U87Ctrl, U87dn and U87Δ899 cells subjected or not to tunicamycin treatment for 16 h. The reference value (1.00) corresponds to the value obtained with U87wt cells in the absence of tunicamycin. Results were normalized using HPRT1 mRNA as standard. qPCR was performed in triplicate on three independent RNA preparations. Data are presented as mean ± SD. **(d)** IRE1 kinase autophosphorylation in U87Δ899 cells. Immunoblotting analysis of total IRE1α (IRE1) and of phospho-Ser724-IRE1 (p-IRE1) proteins after a 2h-incubation with or without tunicamycin.

The relationship identified between IRE1α invalidation and the decrease in EREG mRNA level was further monitored in U87 glioma cells incubated with tunicamycin, an antibiotic that inhibits N-linked protein glycosylation and triggers ER-stress. In order to assess the respective effects of the protein kinase and RNase cytoplasmic domains of IRE1α on EREG expression, we designed an IRE1α mutant (IRE1Δ899) truncated by 78 amino acids at the C-terminal and invalidated for RNase activity (Figure 4a). Three cell clones (R2, R3 and R7) were selected for their expression of the artificial IRE1α isoform and inhibition of ≥ 90% of XBP1 pre-messenger splicing under tunicamycin treatment (Figure 4b). Low levels of MIST1 transcripts were consistently detected in U87Δ899 cells (Figure 4c), in keeping with the fact that MIST1 is a target gene of the mature XBP1 transcription factor [29]. Conversely, IRE1α autophosphorylation (phospho-Ser724-IRE1) was still effective in U87Δ899 clones and was upregulated with tunicamycin (Figure 4d). Thus, the IRE1Δ899 construct acts as a selective dominant-negative mutant of IRE1 RNase and does not notably affect IRE1 kinase activity.

**Figure 5:**
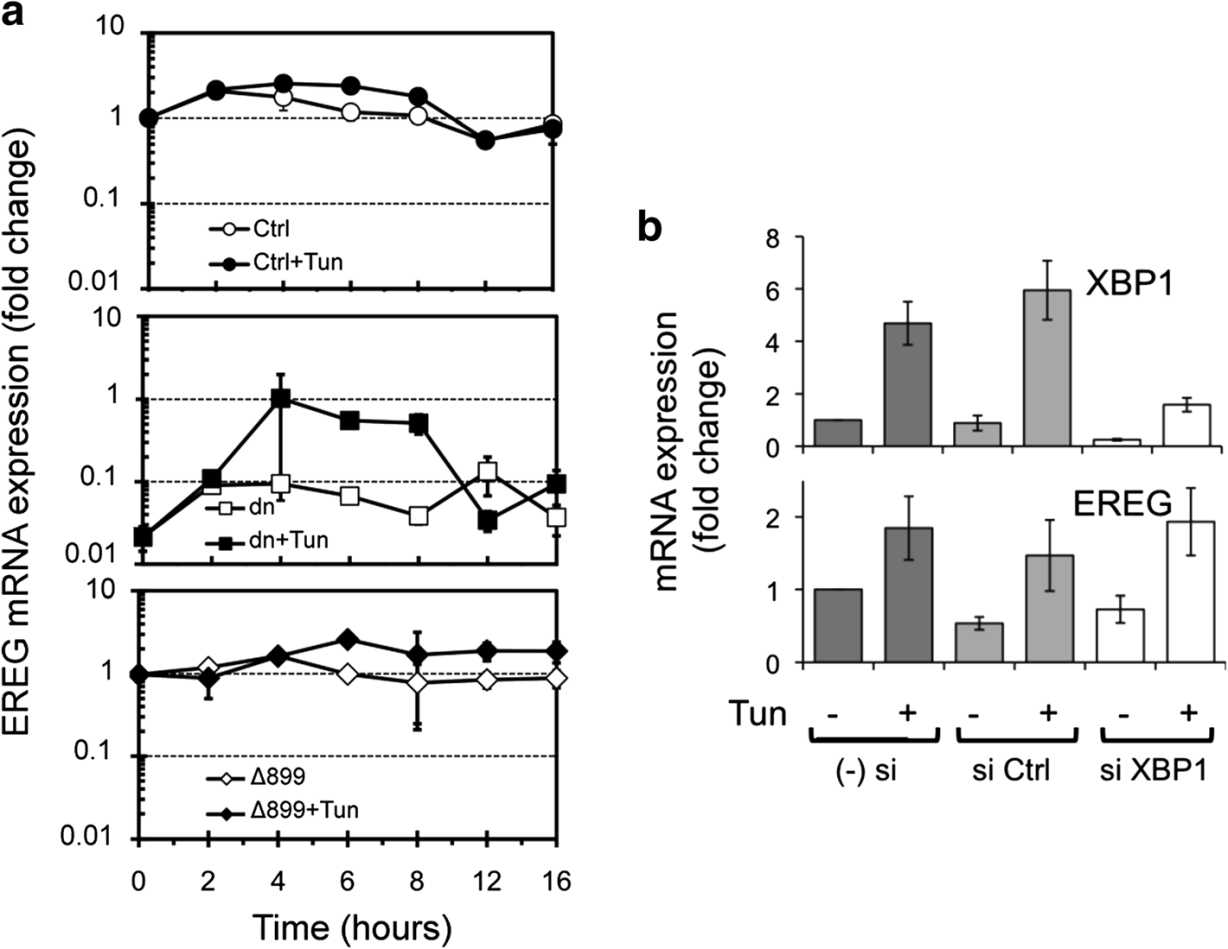
**EREG mRNA expression in U87 cells is independent of IRE1 RNAse activity and of XBP1. (a)** Kinetic analysis of the expression of EREG transcripts by U87Ctrl, U87dn and U87Δ899 cells with or without tunicamycin (Tun). qPCR values were presented as fold-increase relative to the reference value obtained in U87Ctrl cells at the beginning of the experiment (t = 0). HPRT1 was used as the internal standard and values are represented as the mean of triplicate experiments ± SD. **(b)** siRNA knockdown experiments. mRNA expression of XBP1 and EREG in XBP1 siRNA-transfected (si XBP1), nontarget siRNA-transfected cells (si Ctrl) or in untransfected U87wt cells (− si). After transfection, U87wt cells were incubated for 6 h with or without 10 μg/ml tunicamycin. Presence of mRNA was monitored by qPCR. Results were expressed as fold-change relative to untransfected U87 cells without tunicamycin and were normalized using HPRT1 mRNA detection.

Kinetic expression of EREG was analyzed in U87 cell mutants. EREG mRNA levels were similar in U87Ctrl and in U87Δ899 cells in basal conditions and were transiently and modestly (~2.5-fold) increased in the two cell variants in response to either tunicamycin (Figure 5a) or thapsigargin (not shown) treatments. Again, U87dn mutant cells defective in both IRE1 kinase and IRE1 RNase activities produced much lower amounts of EREG under basal condition, a partial recovery of EREG transcript accumulation being observed after 4 to 8 h of incubation with tunicamycin (Figure 5a). Thus, invalidation of IRE1 RNase activity did not compromise EREG expression whereas the absence of both kinase and RNase functions strongly affected its production. siXBP1 knockdown, which achieved significant silencing of the XBP1 gene, confirmed that EREG expression was independent of the IRE1 RNase/XBP1 axis (Figure 5b).

**Figure 6:**
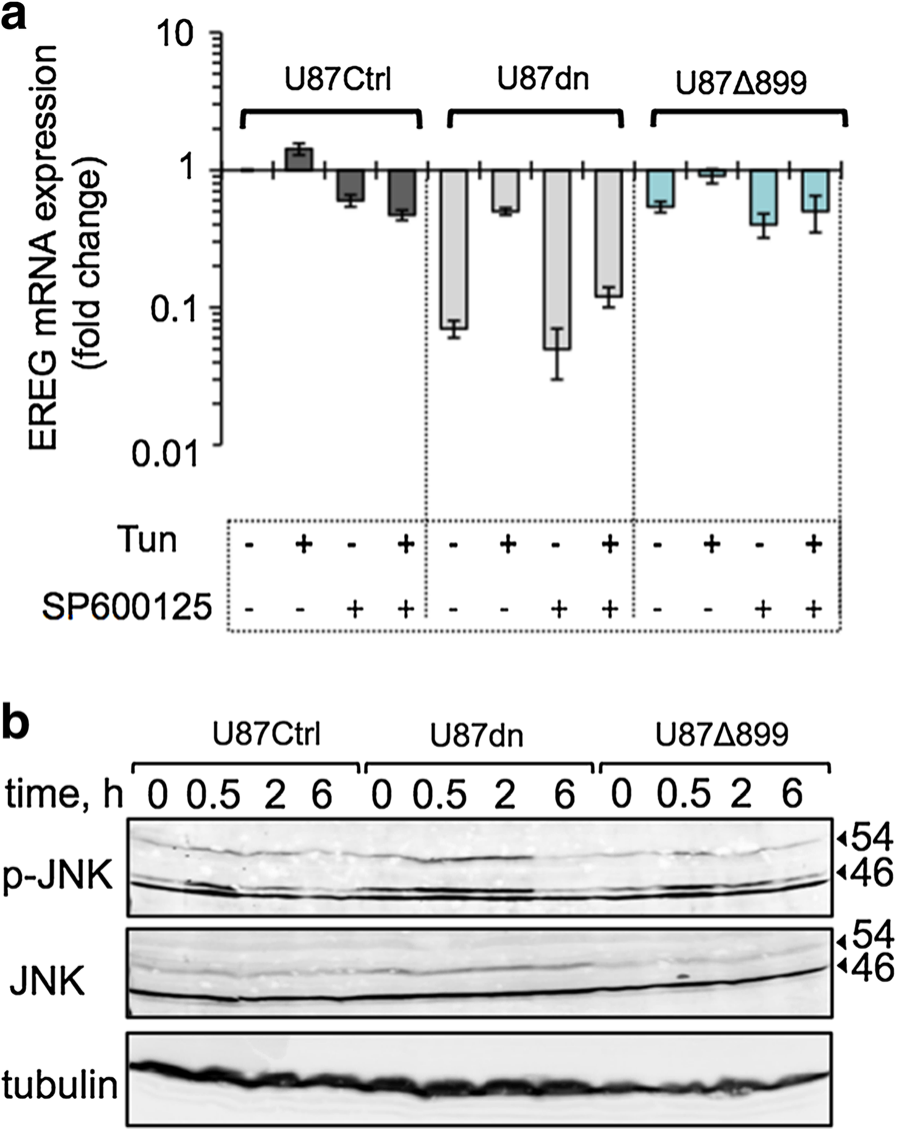
**Effect of the pan-JNK inhibitor SP600125 on EREG expression. (a)** EREG transcript level was measured by qPCR in U87 cells after a 6-h incubation with or without 10 μg/ml tunicamycin and/or 25 μM SP600125. Results were expressed as fold-change relative to U87Ctrl cells in the absence of Tun and SP600125 and were normalized using the HPRT1 reference gene. Results are mean values ± SD. **(b)** Kinetics of JNK phosphorylation in the presence of 10 μg/ml tunicamycin. U87 cells were treated with or without Tun as above. Cell extracts were used for immunoblotting to measure activation of JNK using an anti-phospho-JNK (p-JNK) antibody and antibodies directed against the total (JNK) protein. α-tubulin was used as the reference.

Since JNK activation can be controlled by IRE1α kinase activity [30], we further investigated EREG production in the presence of the specific pan-JNK inhibitor SP600125. Notably, inhibition of JNK compromises tunicamycin-mediated induction of EREG in both U87Ctrl and U87Δ899 cells after 6h of incubation (Figure 6a). Thus, involvement of the JNK pathway for IRE1-dependent regulation of EREG was irrespective of the IRE1 RNase status. Moreover, tunicamycin partially restored the ability of U87dn cells to accumulate EREG transcripts and this inducible effect was also strongly hindered by treatment with SP600125. Thus, both IRE1-dependent and IRE1-independent pathways may converge in U87 cells toward JNK signaling and EREG expression under tunicamycin treatment. This is also consistent with the fact that JNK phosphorylation was increased by tunicamycin in all cell variants, including U87dn cells (Figure 6b).

## Discussion

EREG is a member of the EGF-like growth factor family acting through ErbB tyrosine-kinase receptors and functionnally associated to cell proliferation, survival and migration of a wide range of cell types [2,12,31,32]. Its reported functions in mammals include tissue protection, role in development, reproduction, tissue repair and immune-related responses [33-36]. EREG protein is synthesized as a 163 amino-acid transmembrane precursor and is converted to a diffusible peptide by proteolytic cleavage [12]. Its activities require binding to ErbB1 or ErbB4 transmembrane receptors and transduction signaling through their dimeric combinations with any members of the ErbB family [2,13].

Increased expression of EREG was associated to carcinoma growth, invasion and angiogenesis [16,19,20,23,37] and correlated with poor prognosis [18]. However, the possible implication of EREG in glioma development has not yet been addressed, even though the pathological significance of EGFR has been well established in this pathology. High numbers of wild type or mutated ErbB1 receptors were often detected in primary glioblastomas and in WHO grade II and III oligodendrogliomas [3,4]. The upregulation of the three other ErbB family members in malignant glioma has also been documented [4,5,7].

In this work, EREG expression analyses were performed in several glioma cell lines and were also inventoried in high-grade gliomas from the GEO and Oncomine databases. Both practical and database approaches led to convergent results and indicated that gliomas, as reported for breast cancers [16], produced EREG in highly variable amounts. Same disparities were also observed in gliomas when considering other EGF-like peptides [9,10]. The reasons underlying these modulations likely reflect the wide heterogeneity of gliomas and the possible intervention of a set of transcription factors involved in EREG expression and tumor progression [38-44].

**Figure 7:**
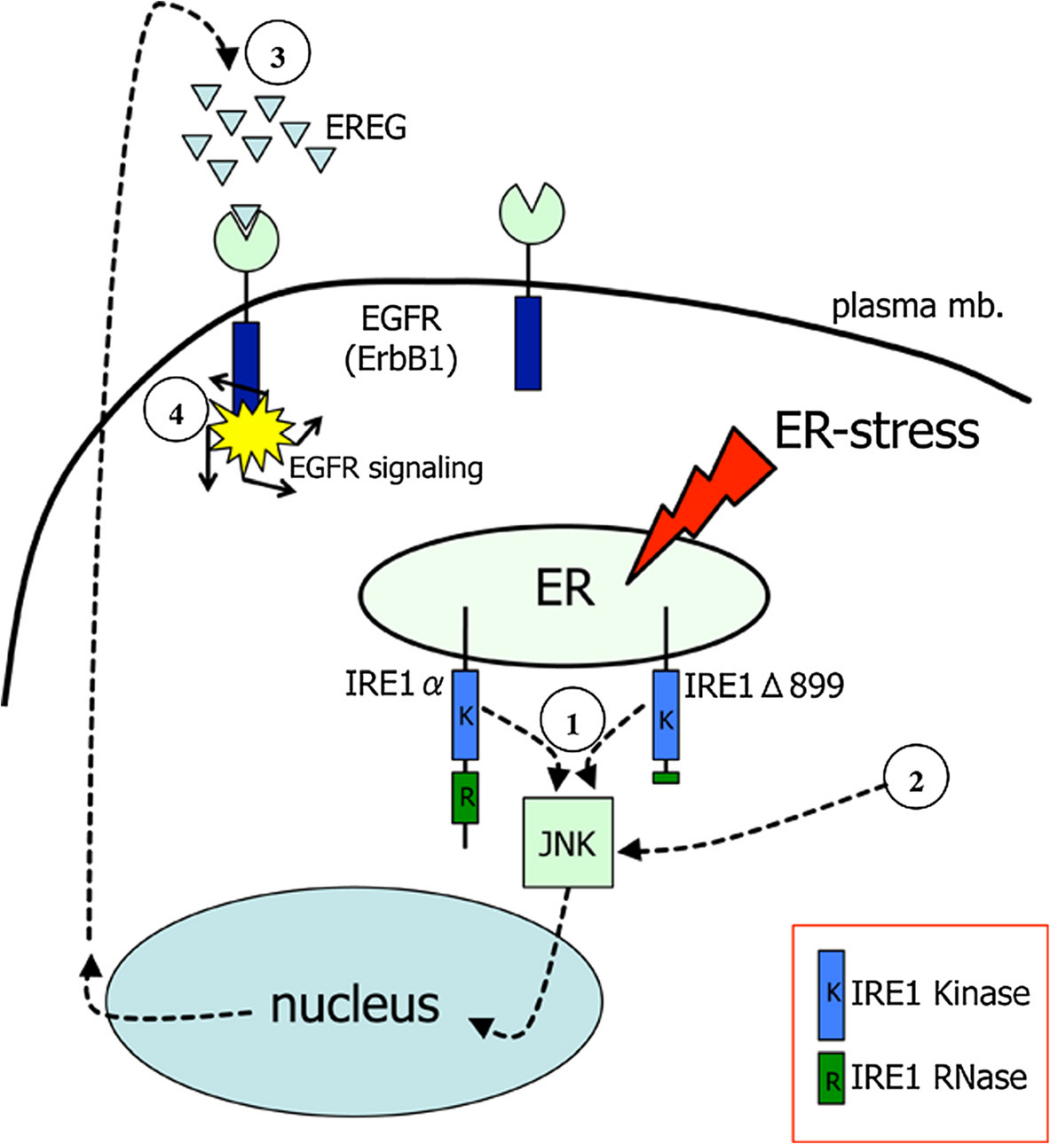
**Proposed scheme depicting the relationship between IRE1α and the autocrine loop mediated by EREG through ErbB1.** IRE1α is a transmembrane protein of the endoplasmic reticulum (ER) and an upstream activator of the JNK to EGFR signaling ①. IRE1 kinase, but not the IRE1 RNase domain, contributes to the high level of EREG production in these cells. Treatment of the cells with the UPR inducers tunicamycin and thapsigargin increases EREG expression using both IRE1α-dependent ① and IRE1α-independent ② pathways. EREG secretion leads to the activation (③ and ④) of EGFR, a protein constitutively expressed by U87 cells. The resulting effect is the autocrine activation of cell proliferation and migration. HB-EGF is another ligand of EGFR and is also expressed by U87 cells. Endogenous expression of EREG and HB-EGF provides a rationale for the consistent level of EGFR autophosphorylation observed under basal conditions. EREG-mediated autocrine loop and sustained activation of EGFR potentially contribute in glioma initiation and progression.

We also showed that the U87 glioma cell line expressed EREG under the dependence of the UPR sensor IRE1α. Inhibition of IRE1α activity, either conducted at the mRNA (siRNA knockdown) or protein (dominant-negative strategy) levels, down-regulated EREG transcript accumulation. In addition, chemical inducers of the UPR such as thapsigargin [45], tunicamycin (this work) or Npi-0052 [46], promote EREG mRNA accumulation in cells, which again suggest a functional link between ER-dependent signaling and EREG expression (see Figure 7, summary illustration).

IRE1α is a bifunctional kinase/RNase enzyme. We evaluated the possible contribution of IRE1 RNase to EREG expression by using a C-terminal truncated IRE1α mutant whose production in cells led to RNase inhibition while maintaining IRE1α autophosphorylation capabilities. Using this mutant, we observed that EREG was expressed at similar rate in RNase-deficient cells as in control cells. In addition, siRNA-mediated knockdown of XBP1 had no significant impact on EREG transcript levels. Thus, the high production of EREG in U87 cells is subordinated to the presence of IRE1α but is not significantly affected after blockade of either IRE1 RNase or XBP1 functions.

Since IRE1 kinase activity is an upstream mediator of JNK signaling [30], we used the pan-JNK inhibitor SP600125 in order to examine the possible involvement of the IRE1/JNK transduction pathway as an alternative to the IRE1 RNase-dependent axis for production of EREG. The two pathways can be functionally dissociated [47,48], which is consistent with the fact that IRE1α autophosphorylation status in U87 cells does not strictly correlated with the IRE1 RNase-mediated splicing of pre-XBP1 mRNA [22]. As reported here, SP600125 decreased EREG mRNA expression in wild type cells and in cells selectively blocked for IRE1 RNase activity, suggesting that both the IRE1 kinase domain and JNK contributed to EREG expression. Two transcription factors activated downstream of JNK signaling (egr-1 and c-jun [38,44,49]) were found to modulate EREG expression thus providing a possible molecular link between activation of IRE1α and EREG expression. Interestingly, we showed that U87dn cells expressing low to undectable amounts of IRE1α also responded to tunicamycin treatment by increasing JNK phosphorylation and EREG mRNA accumulation. Therefore, IRE1-independent pathways may also converge on EREG expression through JNK signaling. Several possible explanations may support this result, including the existence of secondary stimulatory loops mediated by cytokines production independently of the UPR [49,50].

U87 cells release EREG in high amounts and selectively co-express ErbB1 and ErbB2 proteins, but not ErbB3 and ErbB4 proteins. The presence of an autocrine loop mediated by EREG through ErbB1 was demonstrated by the fact that anti-ErbB1 and anti-EREG antibodies (but not anti-ErbB2 antibodies) reduced the basal cell proliferation rate in culture, which was not observed in IRE1α-deficient cells underexpressing EREG. Such an autocrine effect mediated by EREG has also been reported in normal cells [31,32]. In addition, other EGF-like ligands such as TGFα and HB-EGF are involved in self-activation loops in gliomas producing ErbB1 [2,9-11].

## Conclusion

Our data strongly support the view that autostimulatory effects involving EREG expression under the control of IRE1α may be expected in different subtypes of gliomas. Over-production of EREG may occasionally contribute to glioma cell growth and migration as well as to secondary effects in brain cancer pathology, including vascular remodeling and reactive gliosis [23,51].

## Abbreviations

CAM: Chorio-allantoic membrane; EREG: Epiregulin; HB-EGF: Heparin-binding epidermal growth factor-like growth factor; HPRT: Hypoxanthine-guanine phosphoribosyl transferase; IRE1: Inositol-requiring enzyme 1; LCM: Laser Capture Microdissection; SPARC: Secreted protein acidic and rich in cysteine; THBS: Thrombospondin.

## Competing interests

The authors declare no conflict of interest. Roche and Merck Serono were informed of the results of the study but did not contribute to any phase of the study design, analysis, interpretation of the data, or writing of the manuscript.

## Authors' contributions

GA, AJ, SG, OM and MM designed the research; DM, OM and NP performed cDNA cloning and subcloning; GA, AJ, MD, SG, DM, OM, SN and MM carried out cell culture, qPCR and protein expression analyses; GA and AJ developed siRNA experiments; GA, AJ, MD, RP performed *in vivo* experiments; MMaitre and AF performed laser microdissection experiments; PV, MS, AB, OM and MM provided unique research material and analytic tools; GA, AJ, MD, SG, RP, SN, MMaitre, MS, DM, OM and MM analyzed data; OM and MM wrote the paper and the other authors critically reviewed the manuscript. The final version of the manuscript was approved by all authors.

## Pre-publication history

The pre-publication history for this paper can be accessed here:

http://www.biomedcentral.com/1471-2407/13/597/prepub

## Supplementary Material

Additional file 1 Primers used in this study. Click here for file

Additional file 2 **Kaplan-Meier survival analysis of mice bearing U87Ctrl brain tumors and treated with Erbitux®.** Mice implanted in brain with U87Ctrl cells were treated three times a week from day 4 to day 32 after implantation either with 400 μg/ml of anti-human EGFR antibody (Erbitux®) or with PBS (n = 9). Click here for file

Additional file 3 **EREG mRNA expression in glioma: a survey of the literature.** (a) Reports of EREG expression in cells and tissues as depicted in GEO Omnibus (http://www.ncbi.nlm.nih.gov/geo/). (b) Analysis of the Oncomine database (http://www.oncomine.org) for the modulation of expression of EREG transcript in malignant glioma. Click here for file
