# Supplementary material for: High epiregulin expression in human U87 glioma cells relies on IRE1α and promotes autocrine growth through EGF receptor
Source: BMC Cancer. 2013 Dec 13;13:597. doi: 10.1186/1471-2407-13-597 (PMC3878670; doi:10.1186/1471-2407-13-597)
Supplement: Additional file 1 — Primers used in this study. [file 1471-2407-13-597-S1.pdf]

gene	forward primer (5'->3')	reverse primer (5'->3')
β-actin	CGTACCACTGGCATCGTGAT	GTGTTGGCGTACAGGTCTTT
ErbB1	CCACCAAATTAGCCTGGACA	CGCGACCCTTAGGTATTCTG
ErbB2	AGGGAAAACACATCCCCCAAA	CGGACATGGTCTAAGAGGCA
HPRT1	CCAGACAAGTTTGTTGTAGG	TCCAAACTCAACTTGAACTC
Epiregulin	GCTCTGACATGAATGGCTATTGT	TGTTCACATCGGACACCAGTAT
HB-EGF	CCCTCCCACTGTATCCACG	AGTGACTCTCAAAAGGTCCAGA
MIST1	CGGACAAGAAGCTCTCCAAG	CTGGACATGGTCAGGATGGT
S16	GTCACGTGGCCCAGATTTAT	TCTCCTTCTTGGAAGCCTCA
SPARC	GAGAGCGCGCTCTGCCTGCCG	CACCACCTCTGTCTCATCAGGC
THBS1	TGCCTGATGACAAGTTCCAAG	CCAGAGTGGTCTTTCCGCTC
α-tubulin	GAGTGCATCTCCATCC	TAGAGCTCCCAGCAGGCATT
XBP1	CTGGAACAGCAAGTGGTAGA	CTCCTCCAGGCTGGCAGG
