# Supplementary figures and images for: High epiregulin expression in human U87 glioma cells relies on IRE1α and promotes autocrine growth through EGF receptor
Source: BMC Cancer. 2013 Dec 13;13:597. doi: 10.1186/1471-2407-13-597 (PMC3878670; doi:10.1186/1471-2407-13-597)

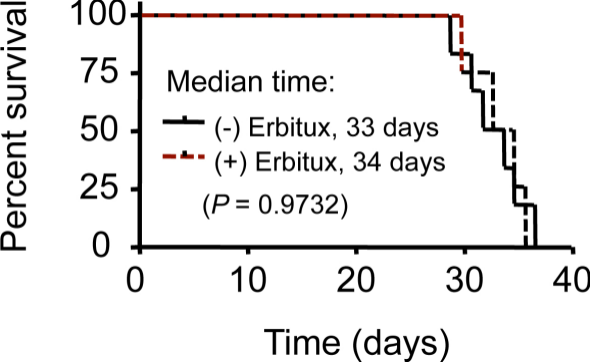

Supplement: Additional file 2 — Kaplan-Meier survival analysis of mice bearing U87Ctrl brain tumors and treated with Erbitux®. Mice implanted in brain with U87Ctrl cells were treated three times a week from day 4 to day 32 after implantation either with 400 μg/ml of anti-human EGFR antibody (Erbitux®) or with PBS (n = 9). [file 1471-2407-13-597-S2.pdf]
