# Supplementary material for: High epiregulin expression in human U87 glioma cells relies on IRE1α and promotes autocrine growth through EGF receptor
Source: BMC Cancer. 2013 Dec 13;13:597. doi: 10.1186/1471-2407-13-597 (PMC3878670; doi:10.1186/1471-2407-13-597)
Supplement: Additional file 3 — EREG mRNA expression in glioma: a survey of the literature. (a) Reports of EREG expression in cells and tissues as depicted in GEO Omnibus (http://www.ncbi.nlm.nih.gov/geo/). (b) Analysis of the Oncomine database (http://www.oncomine.org) for the modulation of expression of EREG transcript in malignant glioma. [file 1471-2407-13-597-S3.zip › 2097470492962767_add2/2097470492962767_add3.pdf]

ligand	Number and type of samples	Grade	GEO accession number	ref
EREG	6 GBM cell lines	IV	GDS2728/GSE7181	(Beier *et al*., 2007)
	50 gliomas	I to IV	GDS1813/GSE2223	(Bredel *et al*., 2005)
	180 gliomas	II to IV	GDS1962/GSE4290	(Sun *et al*., 2006)
	100 malignant gliomas	III and IV	GDS1815/GSE4271	(Costa *et al*., 2010; Phillips *et al*., 2006)

Table S2a: Transcriptome analysis reporting EREG gene expression in low- and high-grade human gliomas as depicted in the Gene Expresssion Omnibus (GEO) database.

References:

Beier D, Hau P, Proescholdt M, Lohmeier A, Wischhusen J, Oefner PJ *et al*. (2007). CD133(+) and CD133(-) glioblastoma-derived cancer stem cells show differential growth characteristics and molecular profiles. *Cancer Res* **67:** 4010-4015.

Bredel M, Bredel C, Juric D, Harsh GR, Vogel H, Recht LD *et al*. (2005). Functional network analysis reveals extended gliomagenesis pathway maps and three novel MYC-interacting genes in human gliomas. *Cancer Res* **65:** 8679-8689.

Costa BM, Smith JS, Chen Y, Chen J, Phillips HS, Aldape KD *et al*. (2010). Reversing HOXA9 oncogene activation by PI3K inhibition: epigenetic mechanism and prognostic significance in human glioblastoma. *Cancer Res* **70:** 453-462.

Phillips HS, Kharbanda S, Chen R, Forrest WF, Soriano RH, Wu TD *et al*. (2006). Molecular subclasses of high-grade glioma predict prognosis, delineate a pattern of disease progression, and resemble stages in neurogenesis. *Cancer Cell* **9:** 157-173.

Sun L, Hui AM, Su Q, Vortmeyer A, Kotliarov Y, Pastorino S *et al*. (2006). Neuronal and glioma-derived stem cell factor induces angiogenesis within the brain. *Cancer Cell* **9:** 287-300.
