# Supplementary material for: High epiregulin expression in human U87 glioma cells relies on IRE1α and promotes autocrine growth through EGF receptor
Source: BMC Cancer. 2013 Dec 13;13:597. doi: 10.1186/1471-2407-13-597 (PMC3878670; doi:10.1186/1471-2407-13-597)
Supplement: Additional file 3 — EREG mRNA expression in glioma: a survey of the literature. (a) Reports of EREG expression in cells and tissues as depicted in GEO Omnibus (http://www.ncbi.nlm.nih.gov/geo/). (b) Analysis of the Oncomine database (http://www.oncomine.org) for the modulation of expression of EREG transcript in malignant glioma. [file 1471-2407-13-597-S3.zip › 2097470492962767_add2/2097470492962767_add4.pdf]

Table S2b: References reported in the Oncomine analysis for EREG comparison between the indicated brain tumors *vs*. normal brain across 26 analyses.

Analyses

1-4:	Anaplastic astrocytoma, anaplastic oligodendroglioma, primary glioblastoma, secondary glioblastoma (Beroukhim et al., 2007).
5-8:	Anaplastic oligoastrocytoma, anaplastic oligodendroglioma, glioblastoma, oligodendroglioma (Bredel et al., 2005).
9-10:	Anaplastic oligoastrocytoma, anaplastic oligodendroglioma (French et al., 2005).
11:	Pilocytic Astrocytoma (Gutmann et al., 2002).
12:	Glioblastoma (Lee et al., 2006).
13-14:	Glioblastoma, oligoastrocytoma (Liang et al., 2005).
15:	Glioblastoma (Murat et al., 2008).
16:	Malignant glioma (Pomeroy et al., 2002).
17:	Astrocytoma (Rickman et al., 2001).
18-20:	Astrocytoma, glioblastoma, oligodendroglioma (Shai et al., 2003).
21-24:	Anaplastic astrocytoma, diffuse astrocytoma, glioblastoma, oligodendroglioma (Sun et al., 2006).
25-26:	Glioblastoma TCGA, No Associated Paper, 2011

References

Beroukhim R, Getz G, Nghiemphu L, Barretina J, Hsueh T, Linhart D *et al*. (2007). Assessing the significance of chromosomal aberrations in cancer: methodology and application to glioma. *Proc Natl Acad Sci U S A* **104:** 20007-20012.

Bredel M, Bredel C, Juric D, Harsh GR, Vogel H, Recht LD *et al*. (2005). Functional network analysis reveals extended gliomagenesis pathway maps and three novel MYC-interacting genes in human gliomas. *Cancer Res* **65:** 8679-8689.

French PJ, Swagemakers SM, Nagel JH, Kouwenhoven MC, Brouwer E, van der Spek P *et al*. (2005). Gene expression profiles associated with treatment response in oligodendrogliomas. *Cancer Res* **65:** 11335-11344.

Gutmann DH, Hedrick NM, Li J, Nagarajan R, Perry A, Watson MA (2002). Comparative gene expression profile analysis of neurofibromatosis 1-associated and sporadic pilocytic astrocytomas. *Cancer Res* **62:** 2085-2091.

Lee J, Kotliarova S, Kotliarov Y, Li A, Su Q, Donin NM *et al*. (2006). Tumor stem cells derived from glioblastomas cultured in bFGF and EGF more closely mirror the phenotype and genotype of primary tumors than do serum-cultured cell lines. *Cancer Cell* **9:** 391-403.

Liang Y, Diehn M, Watson N, Bollen AW, Aldape KD, Nicholas MK *et al*. (2005). Gene expression profiling reveals molecularly and clinically distinct subtypes of glioblastoma multiforme. *Proc Natl Acad Sci U S A* **102:** 5814-5819.

Murat A, Migliavacca E, Gorlia T, Lambiv WL, Shay T, Hamou MF *et al*. (2008). Stem cell-related "self-renewal" signature and high epidermal growth factor receptor expression associated with resistance to concomitant chemoradiotherapy in glioblastoma. *J Clin Oncol* **26:** 3015-3024.

Pomeroy SL, Tamayo P, Gaasenbeek M, Sturla LM, Angelo M, McLaughlin ME *et al*. (2002). Prediction of central nervous system embryonal tumour outcome based on gene expression. *Nature* **415:** 436-442.

Rickman DS, Bobek MP, Misek DE, Kuick R, Blaivas M, Kurnit DM *et al*. (2001). Distinctive molecular profiles of high-grade and low-grade gliomas based on oligonucleotide microarray analysis. *Cancer Res* **61:** 6885-6891.

Shai R, Shi T, Kremen TJ, Horvath S, Liau LM, Cloughesy TF *et al*. (2003). Gene expression profiling identifies molecular subtypes of gliomas. *Oncogene* **22:** 4918-4923.

Sun L, Hui AM, Su Q, Vortmeyer A, Kotliarov Y, Pastorino S *et al*. (2006). Neuronal and glioma-derived stem cell factor induces angiogenesis within the brain. *Cancer Cell* **9:** 287-300.
